# Colony stimulating factor 2 protects the preimplantation bovine embryo from heat shock

**DOI:** 10.1017/S0967199422000508

**Published:** 2022-10-24

**Authors:** Froylan Sosa, Peter J. Hansen

**Affiliations:** Department of Animal Sciences, D.H. Barron Reproductive and Perinatal Biology Research Program, and Genetics Institute, University of Florida, Gainesville, Florida 32611-0910, USA

**Keywords:** Bovine, CSF2, Heat shock, Preimplantation embryo

## Abstract

Heat stress can have severe deleterious effects on embryo development and survival. The present study evaluated whether CSF2 can protect the developmental competence of the bovine embryo following exposure to a heat shock of 41°C at the zygote and morula stages. In the first experiment, putative zygotes and 2-cell embryos were assigned to receive either 10 ng/ml CSF2 or vehicle, and then cultured for 15 h at either 38.5°C or 41°C and then at 38.5°C until day 7.5. Heat shock reduced blastocyst development for embryos treated with vehicle but not for embryos cultured with CSF2. In the second experiment, day 5 embryos (morula) were treated with CSF2 or vehicle and then cultured for 15 h at either 38.5°C or 41°C and then at 38.5°C until day 7.5. Temperature treatment did not affect development to the blastocyst stage and there was no effect of CSF2 treatment or the interaction. Results indicate that CSF2 can reduce the deleterious effects of heat shock at the zygote or two-cell stage when the embryo is transcriptionally inactive.

## Introduction

Heat stress can have deleterious effects on the ability of a female mammal to become pregnant because of disruptions to follicular development, oocyte competence to become fertilized and embryo development and survival (Hansen, 2009, 2019). The early preimplantation embryo is particularly sensitive to heat stress. In the cow, for example, in which rectal temperature is regulated at ~38.5°C, exposure of zygotes to a temperature as low as 40°C reduced the percentage of embryos developing to the blastocyst stage (Sakatani et al., 2012; Ortega et al., 2016). The preimplantation bovine embryo becomes tolerant to the direct inhibitory effects of heat shock as development proceeds. By the morula stage at day 4 or 5 after fertilization, the embryo has heightened resistance to disruption of development by heat shock (Ealy et al., 1993; Edwards and Hansen, 1997; Sakatani et al., 2004). *In vivo*, also, heat stress at day 1 after oestrus compromised subsequent embryonic development, but there was no effect of heat stress at days 3, 5 and 7 (Ealy et al., 1993). Acquisition of thermotolerance may be related to activation of the embryonic genome, which in the cow occurs at the 8-cell stage (Graf et al., 2014).

Resistance of the oocyte and embryo to elevated temperature in the reproductive tract is increased by actions of specific maternally derived cell signalling molecules. Follicular fluid, for example, contains extracellular vesicles that act on the cumulus–oocyte complex to protect the oocyte from elevated culture temperature (Rodrigues et al., 2019). Insulin-like growth factor 1, which is produced locally in the endometrium (Hansen and Tríbulo, 2019), can protect the oocyte (Lima et al., 2017) and morula (Jousan and Hansen, 2004; Bonilla et al., 2011) from elevated culture temperature.

Colony stimulating factor 2 (CSF2) is another maternally derived cell signalling molecule produced by the endometrium that may protect the embryo from heat shock. The gene for this cytokine is transcribed by the endometrium and the protein can be found in uterine fluid during the first 7 days after ovulation (Tríbulo et al., 2018). There are several lines of evidence that CSF2 may act as a survival factor protecting the embryo from environmental conditions that compromise development or survival. Treatment of bovine embryos with CSF2 increased the proportion that develop to the blastocyst stage, especially when overall developmental competence of the embryos is low (Dobbs et al., 2013). Many of the genes whose expression in the blastocyst is regulated by CSF2 are involved in cell death and survival (Zolini et al., 2020). Treatment with CSF2 also reduced the number of apoptotic cells in morula exposed to heat shock (Loureiro et al., 2011) and caused an increase in the number of blastocysts that remained viable in culture after cryopreservation and thawing (Sosa et al., 2020).

The objective of the present study was to determine whether CSF2 can protect developmental competence of the bovine embryo following a heat shock of 41°C. The cytoprotective action of CSF2 was evaluated at two time points in development: the zygote stage, when the embryo is very susceptible to heat shock, and the morula stage, when the deleterious effects of heat shock on development are low.

## Materials and methods

### Production and culture of embryos

Embryos were produced as described by Sosa et al., (2020). Embryos were produced by fertilization for 18 h with frozen–thawed semen pooled from three bulls of various breeds for each replicate; a different pool was used for each replicate. Putative zygotes (i.e. oocytes exposed to sperm) were placed in groups of ~30 in 50-μl drops of a medium called synthetic oviduct fluid, bovine embryo 2 (SOF-BE2) covered with mineral oil, and cultured in a benchtop incubator (WTA, Cravinhos, SP, Brazil) in a humidified atmosphere of 5% CO_2_, 5% O_2_, 90% (v/v) N_2_ by volume until day 7.5 after fertilization. Embryos were cultured at 38.5°C, the normal body temperature of the cow, unless otherwise stated.

### Experiments

The first experiment was conducted to test whether CSF2 would protect zygotes and two-cell embryos from heat shock. At 26 h after insemination, drops of embryos were treated randomly with either 10 ng/ml CSF2 or vehicle (prepared as described by Sosa et al., 2020) and then cultured for 15 h at either 38.5 or 41.0°C in an humidified atmosphere of 5% CO_2_, 5% O_2_, 90% N_2_. Thereafter, all drops of embryos were cultured at 38.5°C in the same atmosphere until day 7.5 after insemination. Embryos remained in the same drops throughout culture. Cleavage of oocytes was recorded at 72 h after insemination and number of blastocysts at day 7.5. The experiment was conducted in four replicates: the total number of putative zygotes was 298 for vehicle/38.5°C, 277 for CSF2/38.5°C; 278 for vehicle/41°C and 292 for CSF2/41°C.

In the second experiment, embryos were cultured until day 5 after insemination at 38.5°C in an humidified atmosphere of 5% CO_2_, 5% O_2_, and 90% N_2_ At this time, each drop of embryos was randomly treated with either 10 ng/ml CSF2 or vehicle and then cultured for 15 h at either 38.5 or 41.0°C in 5% CO_2_, 5% O_2_, 90% N_2_. After temperature treatment, all embryos were cultured at 38.5°C in the same original drops until day 7.5 after insemination when blastocyst development was measured. In total, four replicates were performed and the total numbers of putative zygotes were 228 for vehicle/38.5°C, 225 for CSF2/38.5°C, 225 for vehicle/41°C and 230 for CSF2/41°C.

### Statistical analysis

Data were analyzed by logistic regression fitted to a binomial distribution using the GLIMMIX procedure of the Statistical Analysis System version 9.4 (SAS Institute, Cary, NC, USA). The mathematical model included effects of temperature (38.5 vs 41.0°C), treatment (vehicle vs CSF2) and the interaction as fixed effects and with replicate as a random effect. Tukey’s test was used to determine the differences between individual means.

## Results

**Figure 1.**
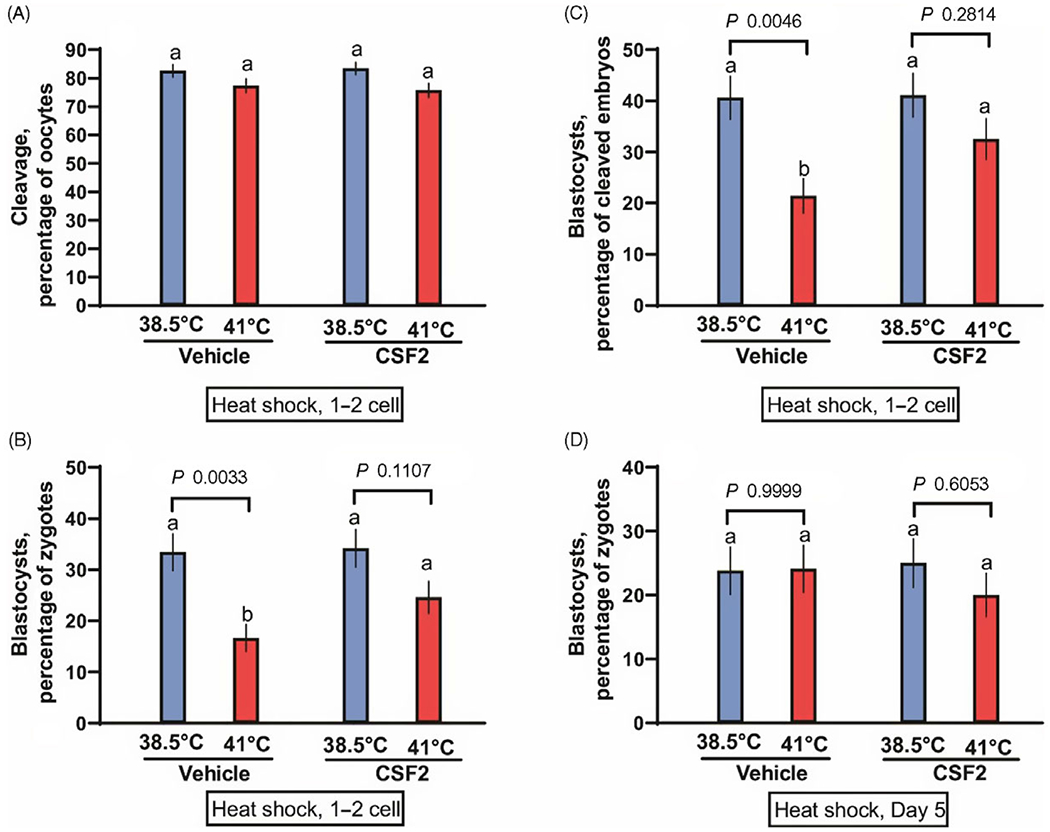
Interactions between heat shock and colony stimulating factor 2 (CSF2) on development of bovine embryos. Embryos were either heat shocked at 41°C for 15 h beginning at 26 h after insemination, when embryos were primarily zygotes or two-cell embryos (A–C) or at day 5 after insemination, when embryos were developed up to the morula stage (D). Means that differ significantly are indicated by different letters above each bar. The probability values for the comparison of 38.5 vs 41.0°C for embryos treated with vehicle and CSF2 are shown above each set of bars.

The first experiment tested whether CSF2 can protect zygotes and two-cell embryos from heat shock. Presumptive zygotes were exposed to a heat shock of 41°C for 15 h. There was no effect of temperature (*P* = 0.9311), CSF2 treatment (*P* = 0.2254) or the interaction (*P* = 0.6998) on the percentage of oocytes that cleaved (Figure 1A). There was, however, a temperature effect (*P* = 0.0003) on the percentage of putative zygotes becoming blastocysts (Figure 1B), with fewer embryos developing to the blastocyst stage after exposure to 41.0°C than embryos cultured continuously at 38.5°C. Treatment tended to affect the percentage of putative zygotes becoming a blastocyst (*P* = 0.0824). Although the interaction was not significant (*P* = 0.1238), further analysis using Tukey’s test revealed that heat shock reduced blastocyst development for embryos treated with vehicle (*P* = 0.0033) but not for embryos cultured with CSF2 (*P* = 0.1107). Similar results were obtained when evaluating the percentage of cleaved embryos developing to the blastocyst stage (Figure 1C). Development was affected by temperature (*P* = 0.0008), CSF2 treatment (*P* = 0.0647) and the interaction between temperature and treatment (*P* = 0.0841). Furthermore, culture of embryos at 41°C decreased the percentage of cleaved embryos becoming blastocysts for embryos cultured with vehicle (*P* = 0.0046) but not for embryos cultured with CSF2 (*P* = 0.2814). Numerically, the reduction in percentage of cleaved embryos becoming blastocysts was 47% for embryos treated with vehicle and 21% for embryos treated with CSF2. Therefore, CSF2 reduced the deleterious effects of heat shock at the zygote to two-cell stages.

The second experiment was conducted as the first, except that heat shock of 41°C was applied at day 5 of development, when the most advanced embryos were at the morula stage. Also, cleavage was not assessed. Results are shown in Figure 1D. In contrast with results with embryos exposed to heat shock at the zygote and twocell stages, temperature treatment did not affect development to the blastocyst stage (*P* = 0.4837). Similarly, there was no effect of CSF2 treatment (*P* = 0.5408) or the interaction (*P* = 0.4384).

## Discussion

The early preimplantation embryo is very sensitive to disruption of its development by elevated temperature, as shown here and elsewhere (please refer to Hansen, 2019 for review). As shown here, the embryo is protected from elevated temperature to some extent by maternally derived molecules. This conclusion is based on the fact that CSF2 partially mitigated the reduction in development caused by exposure of embryos at 26 h after insemination to 41°C for 15 h.

The other maternally derived molecule that has been shown to protect the bovine embryo from heat shock is IGF1 (Jousan & Hansen, 2004; Bonilla et al., 2011). In contrast with CSF2, however, IGF1 was protective towards embryos > 16 cells in development at day 5 after insemination but was not protective to two-cell embryos exposed to heat shock beginning 28 to 30 h after insemination (Bonilla et al., 2011). The thermoprotective effect of IGF1 on day 5 embryos was only observed when embryos were heat shocked by culture at 42°C. This temperature is higher than would be experienced by most cows exposed to heat stress (Dikmen et al., 2012). No thermoprotective effect of IGF1 on day 5 embryos was observed when a more physiological heat shock of 41°C was applied because, as in the present experiment, day 5 embryos were resistant to the negative effects of 41°C.

The mechanisms that embryos acquire to become more resistant to elevated temperature as they advance through development has not been delineated. However, embryonic genome activation is initiated at the 8-cell stage in cattle. Increased susceptibility of embryos before the eight-cell stage could be because activation of thermoprotective mechanisms dependent on transcription are inactive. Given the lack of embryonic gene expression at the zygote and two-cell stage, the thermoprotective effects of CSF2 must involve changes in cellular function independent of transcription. Other pharmacological agents can also protect embryos before embryonic genome activation, including anthocyanins produced by sweet potato (Sakatani et al., 2007) and the globular protein sericin derived from silkworm (Khatun et al., 2018). Perhaps, CSF2 acts post-transcriptionally to regulate effector actions of cytoprotective molecules such as the heat shock proteins, DNA repair proteins, or anti-apoptotic proteins.

The fact that a heat shock of 41°C was not sufficient to reduce development when applied at day 5 of development means that we did not test the thermoprotective properties of CSF2 at this stage of development. It is likely, however, that CSF2 can also provide thermoprotection at this stage. This is because induction of apoptosis in morula at day 6 of development caused by culture at 42°C for 15 h was reduced by CSF2 (Loureiro et al., 2011). In that experiment, CSF2 did not block the reduction in cell number caused by heat shock and so protection was not complete.
